# Production and Characterization of Carnauba Straw (*Copernicia prunifera*) Biochar: Influence of Wax Removal on Adsorption of Methylene Blue

**DOI:** 10.1021/acsomega.6c00684

**Published:** 2026-04-13

**Authors:** Laryssa Coutinho da Silva, Pedro Queiros Santiago, Eva Furtado de Sousa, Joel Pedrosa Sousa, Samuel L. S. Medeiros, Ricardo E. F. Q. Nogueira, M. Alexsandra S. Rios

**Affiliations:** † Department of Metallurgical and Materials Engineering, Center of Technology, 28121 Federal University of Ceará, Pici Campus, Block 729, 60455-760 Fortaleza, CE, Brazil; ‡ Department of Mechanical Engineering, Center of Technology, Federal University of Ceará, Pici Campus, Block 714, 60455-760 Fortaleza, CE, Brazil; § Department of Geology, Center of Sciences, Federal University of Ceará, Pici Campus, Block 912, 60455-760 Fortaleza, CE, Brazil

## Abstract

Synthetic dyes discharged into aquatic systems pose persistent environmental risks, requiring low-cost and sustainable treatment alternatives. This study clarifies the potential of *Copernicia prunifera* (carnauba) straw residues to produce biochars for methylene blue removal from aqueous media. Two bioadsorbents were prepared: a biochar derived from biomass retaining its natural wax layer (CSB) and another obtained from dewaxed biomass before pyrolysis (DCSB). Both biochars and their feedstocks were characterized through proximate and elemental analyses, PSD, FTIR, XRD, and SEM/EDS to elucidate key physicochemical differences. Dewaxing promoted the formation of a more condensed carbon matrix, reflected in lower O/C and (N+O)/C molar ratios, reduced surface oxidation, and a slightly more compact microstructure. These structural changes enhanced the adsorption performance of DCSB, which exhibited a Langmuir maximum capacity of 328.13 mg g^–1^, surpassing CSB. Equilibrium data were best fitted by the Langmuir model, suggesting monolayer adsorption on relatively homogeneous active sites, whereas adsorption kinetics followed a pseudo-second-order model. These results demonstrate that dewaxing carnauba leaf residues before carbonization improves the adsorptive efficiency of carnauba-derived biochars, highlighting their potential as effective and sustainable materials for dye removal from wastewater.

## Graphical abstract

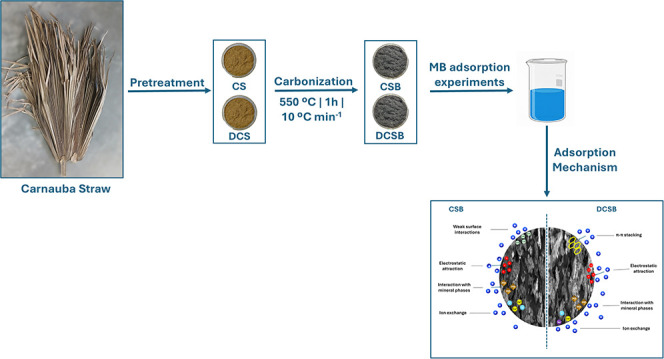

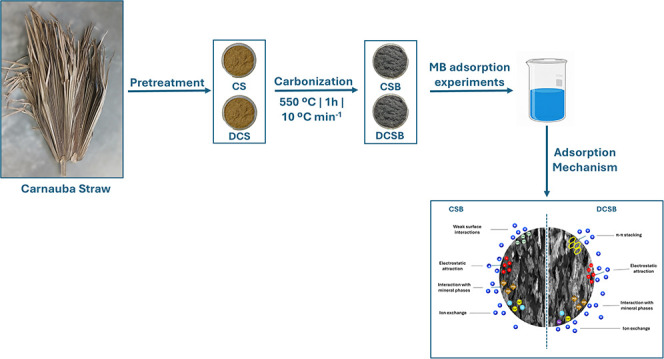

## 1 Introduction

Synthetic dyes are extensively employed in textile, cosmetic, pharmaceutical, and plastic manufacturing, generating effluents that are chemically stable, highly chromatic, and resistant to biodegradation. Among these pollutants, methylene blue (MB) is frequently detected in wastewater due to its widespread industrial use and high persistence. Its release into aquatic environments disrupts photosynthetic activity, alters ecological dynamics, and may pose risks to human health.^1–4^

The mitigation of dye contamination aligns with the United Nations Sustainable Development Goal (SDG) 6 — which focuses on ensuring clean water and sanitation — particularly target 6.3, aimed at reducing pollutant loads and implementing effective, affordable treatment technologies.^5^ Among the available treatment methods, adsorption has emerged as one of the most efficient and versatile techniques due to its operational simplicity, cost-effectiveness, and high removal efficiency for a wide range of dye molecules.^4,6^ Consequently, increasing attention has been directed toward the development of sustainable, low-cost adsorbents.^6^

Biochar is a carbon-rich, porous solid material obtained from the thermochemical decomposition of biomass under oxygen-limited conditions.^7–9^ Although similar in appearance to charcoal, biochar is generated through controlled processes aimed at maximizing carbon retention and minimizing pollutant emissions.^7,10,11^ Its high structural stability, cation exchange capacity, and adsorption potential enable its use in a wide range of applications, including soil fertility improvement, carbon sequestration, environmental remediation, wastewater treatment, pollutant immobilization, and even energy storage.^11–13^

Plant-derived biochars have emerged as a promising bioadsorbent due to their low production cost, the versatility of their surface functional groups, and the opportunity to valorize agricultural residues.^13^ Their physicochemical properties, however, are strongly influenced by the composition of the feedstock and by pretreatment strategies, which can modulate surface chemistry, aromatic condensation, and pore development during carbonization.^4,14^

*Copernicia prunifera* (carnauba) is a native northeastern Brazilian palm exploited for its high-value leaf wax, producing substantial quantities of lignocellulosic residues after wax extraction.^15–19^ Recent declines in wax production, together with the progressive abandonment of carnauba plantations, have raised concerns regarding the economic sustainability of this extractive chain and reinforced the need for new value-added applications for carnauba biomass, particularly within the broader context of bioeconomy and circular resource use.^15,20^

Prior research has investigated the adsorption performance of dewaxed carnauba straw biomass in powder form for the removal of heavy metals and synthetic dyes.^21–23^ However, no studies have examined the conversion of these residues into biochar or evaluated the structural and adsorptive implications of retaining or removing the natural wax layer before pyrolysis. This represents a significant knowledge gap in both biomass valorization and adsorbent design.

Considering this gap, the present study investigates the production and characterization of biochars derived from *C. prunifera* straw with and without their natural wax layer and evaluates their performance in removing methylene blue from aqueous solutions. The feedstocks and resulting biochars were characterized using proximate and ultimate analyses, thermal analysis, FTIR, XRD, PSD, and SEM/EDS. Batch adsorption tests were conducted on biochars to evaluate the effects of contact time, initial dye concentration, and adsorbent dosage. The adsorption mechanism was interpreted through kinetic and equilibrium isotherm modeling.

## 2 Experimental Section

### 2.1 Biomass Preparation

Carnauba straw (dried leaves of *Copernicia prunifera*) was collected from naturally occurring trees on the Pici Campus of the Federal University of Ceará (3.7431° S, 38.5788° W; WGS 84; SISGEN Registration Number A2B4F8E), Fortaleza-Ceará-Brazil. A portion of the straw was shredded and sieved through an 18-mesh screen to obtain the raw carnauba straw sample (CS). Another straw portion had the wax powder layer removed by rubbing with a synthetic sponge before being shredded and sieved through the same mesh, yielding the dewaxed carnauba straw sample (DCS). In industrial contexts, the wax powder is typically removed from carnauba straw either manually, by beating the straw with wooden sticks, or mechanically using beating machines equipped with knife-mill shredders, which simultaneously release the wax powder and fragment the straw.^15^

### 2.2 Biochar Conversion

The CS and DCS samples were subjected to the pyrolysis process. One kg of biomass was used to obtain each biochar. The samples were placed in a ceramic crucible with a lid to limit oxygen availability and maintain oxygen-limited conditions at atmospheric pressure. Subsequently, the samples were heated in a muffle furnace (Q318m21, Quimis, Brazil) at 25 to 550 °C (10 °C/min) for 1 h. After biochar samples (i.e., CSB and DCSB, from CS and DCS, respectively) reached room temperature, they were stored in a desiccator.

### 2.3 Characterization

The biomass and biochar samples were characterized to evaluate the changes induced by pyrolysis and to assess the effect of wax removal on properties relevant to adsorption.

Proximate analysis was conducted according to ASTM D1762-84(2007)^24^ for volatile matter and moisture content, ASTM E1755-01 ^25^ for ashes content. Volatile matter was measured by heating the samples in ceramic at 950 °C for 7 min in a muffle furnace.^24^ Ash content was determined by heating the samples at 575 °C for 3 h.^25^ Moisture content was determined at 105 °C until constant weight using a moisture analyzer (MOC63u, Shimadzu, Japan).^24^ Fixed carbon content was calculated by difference between 100 wt % and the sum of moisture, volatile matter, and ash contents.^9^

Ultimate analysis (C, H, and N) was performed using a CHN elemental analyzer (2400 Series II, PerkinElmer, USA) at the Analytical Center of the Institute of Chemistry (CA-IQ), University of São Paulo (USP). Oxygen content was calculated by difference between 100 wt % and the sum carbon, hydrogen, nitrogen, and ash contents.^9^

Surface morphology and elemental composition were analyzed using a scanning electron microscope (TM3000, Hitachi, Japan) coupled with an energy-dispersive spectroscopy system (ED3000, Swift Analytical, UK). Images were acquired in secondary and backscattered electron modes at an accelerating voltage of 15 kV, and elemental analysis was conducted in the 0–20 keV energy range.

Particle size distribution was determined by laser diffraction using a particle size analyzer (SALD-2300, Shimadzu, Japan) operating with WingSALD II v3.5.0 software, equipped with a flow cell and automatic sampling system. Measurements were performed in aqueous medium with sodium hexametaphosphate (NaHMP) as dispersant, under ultrasonic dispersion for 10 min. The refractive index was adjusted between 1.50–0.01i and 1.70–0.02i, depending on the sample.

X-ray diffraction patterns were collected using a diffractometer (X’Pert PRO, PANalytical, Netherlands) with Co–Kα radiation over a 2θ range of 3–100° and a step size of 0.013°. Fourier-transform infrared (FTIR) spectra were obtained using a spectrometer (VERTEX 70v, Bruker, Germany) equipped with an ATR accessory, operating in the range of 4000–380 cm^–1^ at a resolution of 2 cm^–1^, averaging 128 scans per sample.

Thermogravimetric (TG), differential thermogravimetric (DTG), and differential thermal (DTA) analyses were carried out using a simultaneous thermal analyzer (STA 449 F3 Jupiter, NETZSCH, Germany). Samples were heated from 30 to 900 °C at a rate of 10 K min^–1^ in a controlled atmosphere, using an Al_2_O_3_ crucible. Nitrogen was employed as shielding gas (20 mL min^–1^), and synthetic air (80/20) was used as purge gas (50 mL min^–1^).

### 2.4 Batch Adsorption Experiments

Methylene blue (MB, CAS: 122965-43-9, C_16_H_18_ClN_3_S·*x*H_2_O, MW: 319.85 g mol^–1^) dye was used for batch adsorption studies. A stock solution of 1.0 g L^–1^ was prepared by dissolving 1.0 g of MB in 1.0 L of distilled water. The working solutions of the required concentrations were obtained from the stock solution.

All adsorption experiments were performed in 100 mL glass beakers using a horizontal shaker (SK-180-Pro, Go Shaker, China) at 250 rpm and 25 °C. The solution pH was adjusted to 6.0, since near-neutral conditions favor electrostatic attraction between the biochar surface and the MB molecules, enhancing adsorption efficiency.^26,27^ After each contact time, the suspensions were centrifuged (MIKRO 120, Hettich, Germany) at 4000 rpm for 10 min to separate the supernatant from the solid phase. Table 1 summarizes the conditions used for the batch adsorption experiments.

**Table 1:** Experimental Conditions for Adsorption Studies using CSB and DCSB^a^

**experiment**	**dye concentration**(mg L^–**1**^ **)**	**biochar mass (mg)**	**solution volume (mL)**	**pH** ^b^	**contact time**
**effect of biochar dosage**	15	2, 5, 10,15,20,25	50	6	120 min
**effect of contact time**	15	5			0, 5, 15, 30, 60, 120, 180, 240 min
**effect of initial dye concentration**	2.5, 5, 8, 10, 15, 20, 40, 50, 70, 100, 150	5			120 min

a Adsorption experiments were conducted using both CSB and DCSB under identical conditions.

b Before each experiment, the solution pH was adjusted to 6.0 using 0.1 M HCl or 0.1 M NaOH.

The residual methylene blue concentrations were determined by UV–vis spectrometry (Shimadzu 2600 spectrometer, Shimadzu, Japan), 800–400 nm range, at 664 nm. Each experiment was performed in quadruplicates. The adsorption capacity at equilibrium (*q*_*e*_, mg g^–1^) was calculated according to eq 1:^28,29^

1 $$q_{e}=\frac{\left(C_{o}-C_{e}\right)V}{m}$$

where (*C*_o_, mg L^–1^) is the initial adsorbate concentration, *C*_e_ (mg L^–1^) is the equilibrium concentration, *V* (*L*) is the solution volume, and *m* (*g*) is the adsorbent mass. The removal efficiency (η, (%)) was calculated using eq 2:^28^

2 $$\eta \left(\%\right)=\frac{\left(C_{o}-C_{e}\right)}{C_{o}}\times 100\%$$

The adsorption kinetics were analyzed using the pseudo-first-order (PFO), pseudo-second-order (PSO), Elovich and intraparticle diffusion models. The PFO and PSO model formulas are presented in eq 3 and 4, respectively:^9,28,29^

3 $$\mathrm{PFO}:q_{t}=q_{e}\left(1-\exp\left(-k_{1}t\right)\right)$$

4 $$\mathrm{PSO}:q_{t}=\frac{q_{e}^{2}k_{2}t}{1+q_{e}k_{2}t}$$

where *q*_*t*_ is the uptake at time t in (mg g^–1^), *k*_1_is the PFO constant in (h^–1^) and *k*_2_ (g mg^–1^ h^–1^) is the PSO rate constant. The initial adsorption rate (*h*, *m*g g^–1^ h^–1^) was calculated using eq 5:^9^

5 $$h=k_{2}q_{e}^{2}$$

The Elovich model was applied as expressed in eq 6:^9,28,29^

6 $$q_{t}=\frac{1}{\beta }\ln\left(1+\alpha \beta t\right)$$

where α is the initial sorption rate in (mg g^–1^ h^–1^) and β is the desorption constant in (g mg^–1^). The intraparticle diffusion model proposed by Weber and Morris was evaluated using eq 7:^29^

7 $$q_{t}=k_{p}t^{1/2}+C$$

where *k*_*p*_ is the intraparticle diffusion constant in (mg g^–1^ h^–0.5^) and C is the boundary layer thickness.

Adsorption equilibrium data were fitted using the Langmuir and Freundlich isotherm models. The Langmuir model is expressed by eq 8:^9,28,29^

8 $$q_{e}=\frac{q_{m}K_{L}C_{e}}{1+K_{L}C_{e}}$$

where *q*_*m*_ is the maximum capacity in (mg g^–1^) and *K*_*L*_ *is the* Langmuir constant in (L g^–1^). The dimensionless separation factor (*R*_*L*_), which indicates adsorption favorability, was calculated using eq 9:^9,29^

9 $$R_{L}=\frac{1}{\left[1+K_{L}C_{o}\right]}$$

Adsorption is considered unfavorable when*R*_*L*_ > 1, linear when *R*_*L*_ = 1, favorable when 0 < *R*_*L*_ < 1 is and irreversible when *R*_*L*_ = 0. The Freundlich isotherm model is given by eq 10:^9,28,29^

10 $$q_{e}=K_{F}C_{e}^{\frac{1}{n}}$$

where *K*_*F*_ is the Freundlich’s constant in (mg g^–1^) and 1/*n* is the heterogeneity factor.

## 3 Results and Discussion

### 3.1 Biochar Yield, Bulk Density, and Particle Size Distribution

Biochar yield and other characterization parameters are shown in Table 2. Wax removal did not significantly affect biochar yield (*p* ≥ 0.05). However, it significantly influenced bulk density, with DCS and DCSB exhibiting higher values than CS and CSB, respectively.

**Table 2:** Physicochemical Parameters of the Biomasses and Biochars[Table-fn t2fn1]

**parameters**	**CS**	**DCS**	**CSB**	**DCSB**
**yield (%)**			33.68 ± 1.01^a^	34.27 ± 1.98^a^
**bulk density**(g cm^–3^)	0.2809 ± 0.0012^b^	0.2964 ± 0.0005^a^	0.2553 ± 0.0004^b^	0.2630 ± 0.0010^a^
**particle size distribution (PSD)**				
range (D_90_–D_10_) (μm)	70.29	63.99	52.25	41.17
D_50_ (μm)	34.61	34.87	23.59	27.37
SSA (m^2^ cm^–3^)	0.489	0.374	0.530	0.291
**proximate analysis**				
ash content (%)	12.53 ± 0.31^a^	14.20 ± 0.85^b^	22.73 ± 1.02^a^	28.60 ± 4.30^a^
volatile matter (%)	71.60 ± 1.43^a^	69.90 ± 0.85^a^	35.10 ± 5.25^b^	19.00 ± 3.05^a^
moisture (%)	8.32 ± 0.45^a^	8.97 ± 0.17^a^	3.49 ± 0.30^a^	4.23 ± 0.62^a^
fixed carbon (%)	7.57 ± 1.18^a^	6.97 ± 1.12^a^	38.67 ± 4.71^a^	48.21 ± 7.30^a^
**ultimate analysis and molar ratios**				
C (wt %)	44.66 ± 0.11	42.24 ± 0.30	45.18 ± 0.25	45.63 ± 0.11
H (wt %)	5.29 ± 0.12	4.89 ± 0.14	1.49 ± 0.02	1.65 ± 0.01
N (wt %)	0.88 ± 0.010	0.88 ± 0.02	0.89 ± 0.02	0.92 ± 0.01
O (wt %)	36.66 ± 0.11	37.79 ± 0.46	29.73 ± 0.26	23.21 ± 0.11
H/C	1.41	1.38	0.39	0.43
O/C	0.62	0.67	0.49	0.43
(N+O)/C	0.84	0.92	0.68	0.53
**EDS**				
O (wt %)	35.67	43.01	44.13	47.68
Si (wt %)	22.02	26.82	32.92	34.30
K (wt %)	15.28	6.66	9.39	5.88
Cl (wt %)	17.49	5.74	6.72	2.34
Ca (wt %)	5.04	13.05	4.08	5.29
S (wt %)	2.95	3.34	1.50	2.90
Mg (wt %)	1.58	1.37	1.26	1.25
Na (wt %)				0.36

c CS: raw carnauba straw; DCS: dewaxed carnauba straw; CSB: carnauba straw biochar; DCSB: dewaxed carnauba straw biochar. Values are expressed as mean ± SD when applicable (*n* = 4 for yield, bulk density, and proximate and PSD analyses; *n* = 2 for ultimate analysis). Values with different superscripts (a, b) in the same row and comparison group (CS vs DCS or CSB vs DCSB) are significantly different (*p* ≤ 0.05).

The particle size distribution (PSD) is presented in Table 2. All samples exhibited distinct unimodal distributions. The carbonization process generated biochar particles that were notably finer and more homogeneous, as evidenced by the smaller median particle size (D_50_) and narrower size range (D_90_–D_10_) of CSB and DCSB compared to CS and DCS (Figure 1). The variations in PSD attributable to dewaxing were minor. In contrast, pyrolysis produced more pronounced changes in particle size distribution. This behavior suggests that the shredding and sieving steps effectively standardized the initial particle size of the biomass. More profound structural modifications caused by carbonization were later observed via SEM analysis.

**Figure 1:**
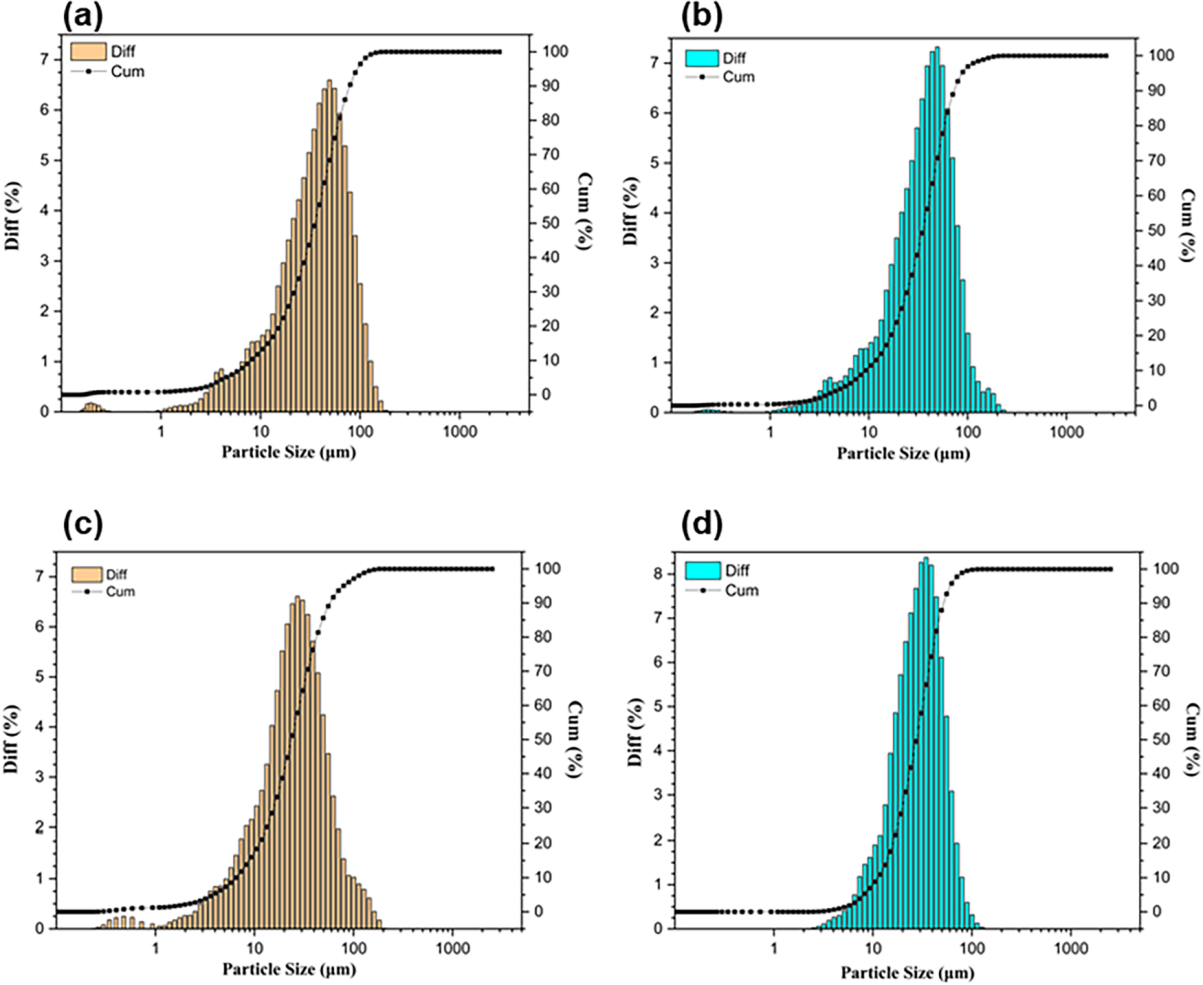
Particle size distribution of biomasses and biochars. (a) CS, (b) DCS, (c) CSB, (d) DCSB. The bars (Diff) represent the differential distribution (relative frequency), and the black lines (Cum) represent the cumulative distribution.

### 3.2 Proximate Analysis

Comparisons between biomass-biochar pair (CS-CSB and DCS-DCSB) confirmed the expected compositional changes induced by pyrolysis — enrichment of ash content and fixed carbon, and the depletion of moisture and volatile matter (Table 2).

The ash content (AC), representing the inorganic phase, was significantly higher (p= 0.023) in DCS compared to CS. This initial difference suggests that the removal of wax fraction effectively concentrated the inherent mineral matter in the DCS sample, leading to a higher inorganic load. After pyrolysis, this trend persisted in the DCSB sample, showing a higher AC than CSB, although this difference was not statistically significant (*p* = 0.07). AC enhances the biochar’s resistance to thermal degradation and provides active sites for adsorption processes.^9,30^

The influence on the organic phases became fully apparent after pyrolysis. In the biomass samples, the volatile matter (VM) and fixed carbon (FC) were not significantly different, indicating the wax’s removal did not drastically alter these proportions before pyrolysis. However, a significant difference (*p* = 0.004) was found between CSB and DCSB in VM, with CSB containing more volatile content than DCSB. VM is released during pyrolysis through thermal cracking of labile organic fractions as condensable vapors and noncondensable gases.^31^ The higher VM in CSB is attributed to the composition of carnauba wax, rich in long-chain fatty esters and hydrocarbons.^32^ During pyrolysis, this labile fraction generates substantial condensable vapors that recondense within the biochar matrix. This interpretation is consistent with the findings of the thermogravimetric analysis.

During pyrolysis, the aliphatic carbon phases of hemicellulose and cellulose are transformed into thermally stable aromatic monomers, which constitute the fixed carbon (FC) fraction of the biochar.^9,30^ The CSB and DCSB did not show a statistically significant difference in terms of FC content (*p* = 0.078). However, the clear trend of a higher mean value in DCSB supports the conclusion that the removal of noncarbonizing wax promoted a more efficient conversion of the structural biomass into stable aromatic carbon, thereby increasing the relative FC yield.

### 3.3 Ultimate Analysis and Molar Ratios

The elemental composition and molar ratios of the biomasses and biochars are shown in Table 2. Carbon, hydrogen, nitrogen, and oxygen are the major elemental constituents of biomasses.^30,33^ Thermal conversion through pyrolysis alters this composition, increasing the C-content while reducing the proportions of H and O due to dehydration, decarboxylation, and decarbonylation reactions.^9,30,34^ These trends are observed in Figure 2a. In contrast, N-content remained constant, indicating retention and reorganization of nitrogen into thermally stable structures that resist volatilization at the applied temperature.^35^

**Figure 2:**
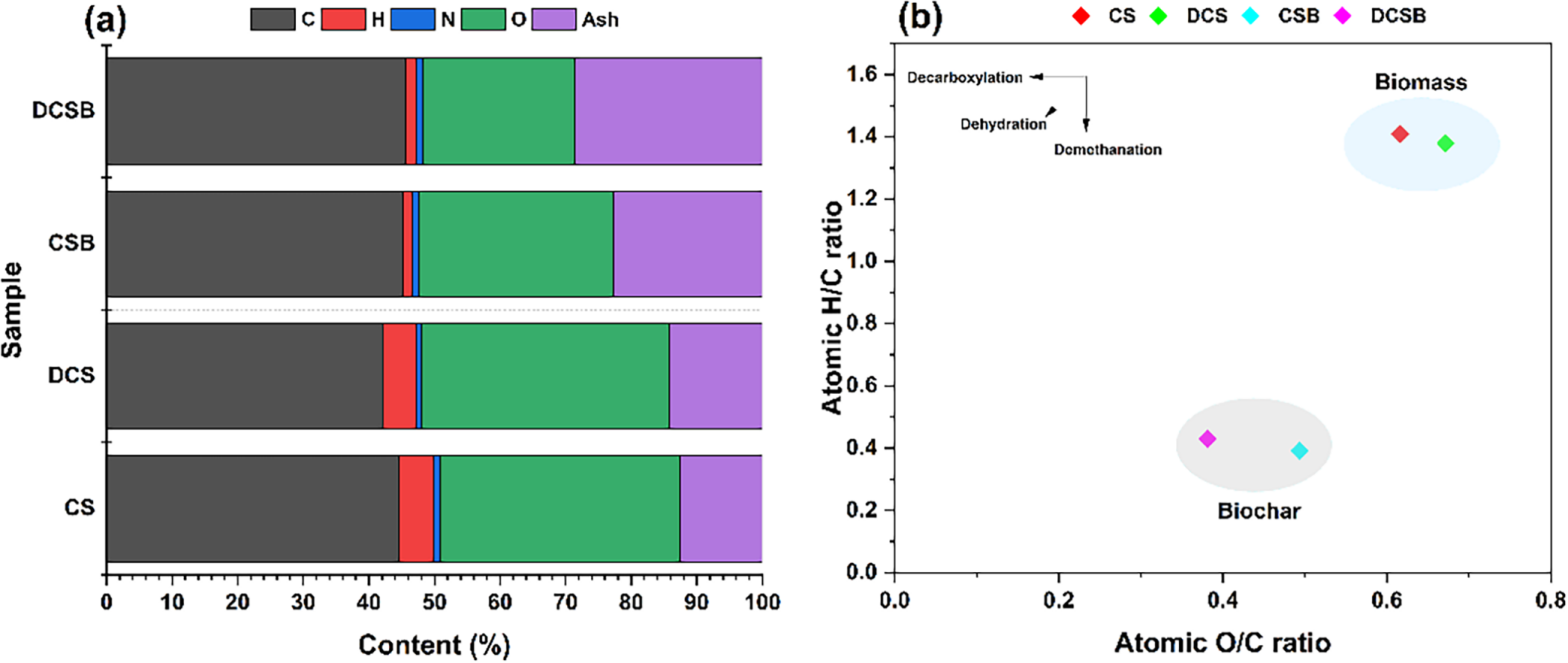
(a) Elemental composition (CHNO) and ash content of the biomasses and biochars. (b) Van Krevelen diagram.

Wax removal decreased the C and H contents and increased the relative O proportion in the biomass (DCS vs CS). After pyrolysis, DCSB exhibited lower O content than CSB. This trend is consistent with the proximate analysis, in which DCSB showed reduced volatile matter and increased fixed carbon, indicating that the absence of the wax fraction influences oxygen release and promotes greater structural condensation during carbonization. In agreement with these compositional changes, the Van Krevelen diagram (Figure 2b) illustrates the structural evolution during carbonization. The H/C ratio decreased markedly after pyrolysis, reflecting the development of highly aromatized carbon domains and increased structural stability.^9,35^ Meanwhile, the O/C ratio followed the trend CSB > DCSB, indicating greater deoxygenation when the wax fraction was removed.

### 3.4 SEM-EDS

The Scanning Electron Microscope (SEM) micrographs of biomasses and biochars produced from carnauba straw are presented in Figure 3a–f. Panels a and c (×30 magnifications) show a general view of CS and DCS morphology, respectively, revealing comminuted yet structurally intact leaf fibers.

**Figure 3:**
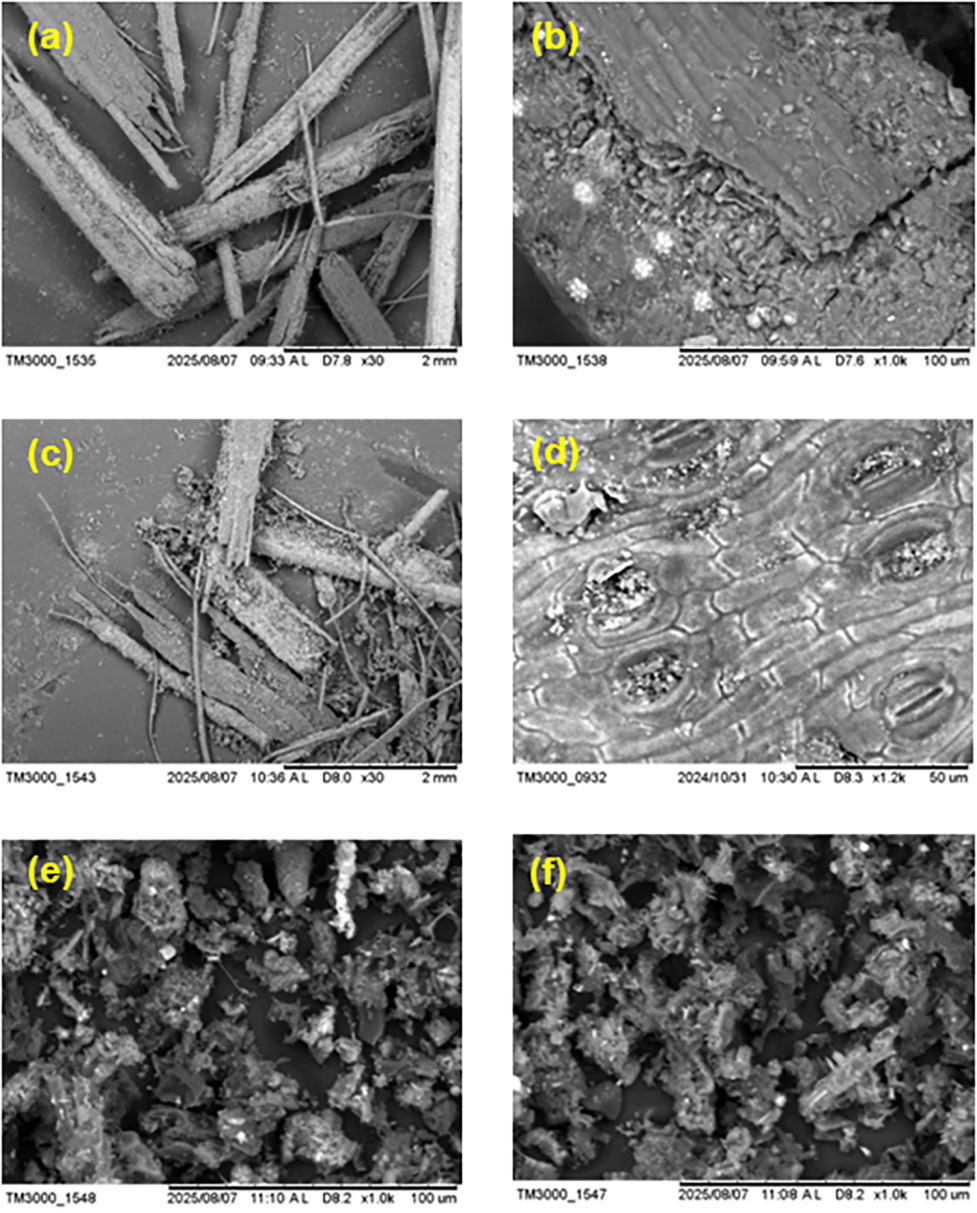
SEM micrographs of the raw biomasses and biochars. (a, b) CS biomass at 30x and 1000× magnification. (c, d) DCS biomass at 30x and 1200× magnification. (e) CSB biochar (1200×). (f) DCSB biochar (1200×).

At × 1000 magnifications, CS exhibits silica accumulations in the form of phytoliths, which are visibly dispersed across the surface (see Figure 3b). These phytoliths are the main form of silicon storage in higher plants and contribute to the mechanical reinforcement and protection of leaf tissues.^36^ Their occurrence agrees with the natural biosilica structures reported for other lignocellulosic biomasses, typically showing heterogeneous and fibrous morphologies.^37^ These silicate structures persisted after pyrolysis, as confirmed by EDS and FTIR analyses, indicating that the inorganic silica framework remains stable under the applied carbonization conditions. At × 1200 magnifications (Figure 3c), DCS exhibits a structure similar to the CS. Preserved stomata are visible, along with a smooth surface displaying horizontally oriented grooves characteristic of the leaf epidermis. Finely dispersed inorganic particulates can also be observed across the surface.

The CSB and DCSB exhibit fragmented and irregular carbonaceous structures at ×1000 magnification (Figure 3d,e), characterized by disrupted plant-derived frameworks and rough surfaces. In both materials, agglomerated and partially collapsed cell-wall residues are visible, forming interconnected voids and irregular cavities. DCSB appears slightly more compact, with denser agglomerates and fewer open gaps, whereas CSB shows a more open and loosely arranged structure.

This morphological difference is consistent with the compositional trends observed in the proximate and elemental analyses. DCSB exhibited a lower volatile matter and a lower O/C ratio, indicating a greater degree of structural condensation during carbonization. The prior removal of the wax fraction likely reduced the contribution of thermally labile compounds and favored the formation of a more condensed aromatic carbon framework. In Figure 3f, this effect is reflected in the more aggregated and compact carbon matrix observed for DCSB compared to CSB, suggesting that the dewaxing step influenced the structural organization of the resulting biochar and the arrangement of surface structures.

According to the EDS results (Table 2), oxygen and silicon were the predominant elements in all samples, confirming the abundance of phytogenic silica structures and their persistence after pyrolysis, as also supported by SEM and FTIR observations. An increase in Si and O contents was observed in both biochars compared with their respective feedstocks, reflecting the relative enrichment of inorganic components due to the loss of organic matter during carbonization. Potassium and chlorine markedly decreased after pyrolysis, likely because of the volatilization of KCl and other soluble salts at elevated temperatures.^30,34^ Calcium remained constant, suggesting its association with thermally stable mineral phases.^28^ Minor amounts of S, Mg, and Na were also detected, corresponding to residual inorganic constituents naturally present in the biomass.^30,34^

### 3.5 XRD

The diffractograms of the biomasses and biochars, as well as the identification of the main crystalline phases, are shown in Figure 4. In the biomasses (CS and DCS), the reflections at ∼20.7 and ∼25° correspond to the semicrystalline cellulose structure,^38,39^ typically associated with the (110) and (200) crystallographic planes of cellulose I.^40,41^ The peak at ∼20.7° is more intense in DCS, while the 25° reflection is sharper in CS. In DCS, it appears with a shoulder at ∼27.6° and a broader amorphous base. Such behavior is consistent with reports showing that hemicellulose, lignin and extractive-rich domains contribute to amorphous scattering, often producing broad halos and partially suppressing or distorting cellulose reflections.^39,41,42^ The broader amorphous region observed in DCS between ∼42 and ∼48°, with only a weak feature near ∼47.5°, is compatible with disordered organic structures and low-crystallinity mineral phases commonly found in untreated or mildly pretreated biomass.^39,41,42^

**Figure 4:**
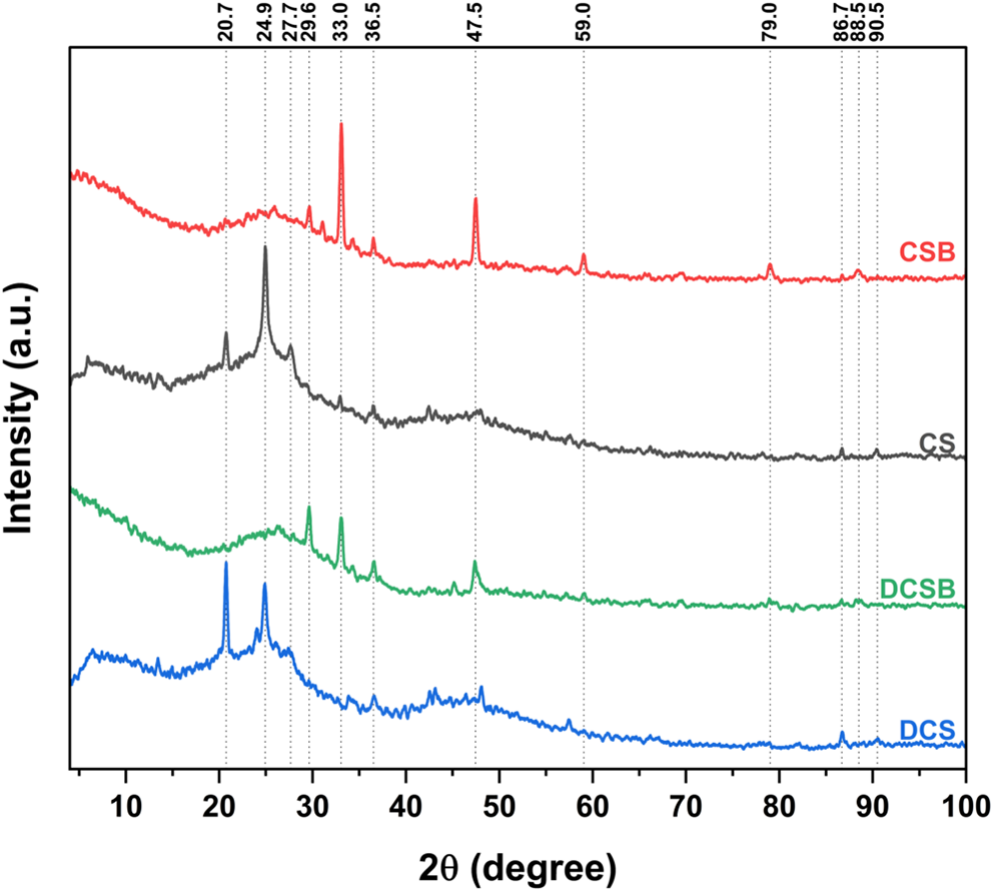
XRD patterns of CS, DCS, CSB and DCSB.

After pyrolysis, CSB and DCSB retain the same mineral phases, but peaks become sharper due to the removal of volatile organic matter. A broad diffraction band between approximately ∼20 and ∼30° can be observed in the biochars, which is commonly attributed to the (002) plane of turbostratic carbon structures, characteristic of partially ordered aromatic carbon layers formed during biomass carbonization.^43^

Reflections attributed to calcite (∼29.7°, corresponding to the (104) plane),^40^ Ca- and K-bearing silicate and phosphate phases (∼33°),^44^ and quartz (secondary reflection near ∼36.5°, related to the (110) plane)^45^ are observed in both biochars. High-angle features at ∼47.5, ∼79, and ∼90.5° are consistent with higher-order reflections of silica, feldspathic aluminosilicates, and Ca/K salts.^4,28,41,44^ Nevertheless, intensities are consistently higher in CSB, which may reflect that wax removal (hydrocarbons and esters) altered the relative contribution of the amorphous organic matrix in DCSB and thus, the relative visibility of mineral crystalline domains^42,46^

### 3.6 FTIR

Figure 5 provides the FTIR spectra of the biomass and biochar samples. The spectra of CS and DCS exhibited bands that are typical of lignocellulosic materials. The broad absorption at ∼3410–3415 cm^–1^ corresponds to O–H stretching, associated with hydroxyl groups in cellulose, hemicellulose, and lignin, as well as adsorbed water.^47,48^

**Figure 5:**
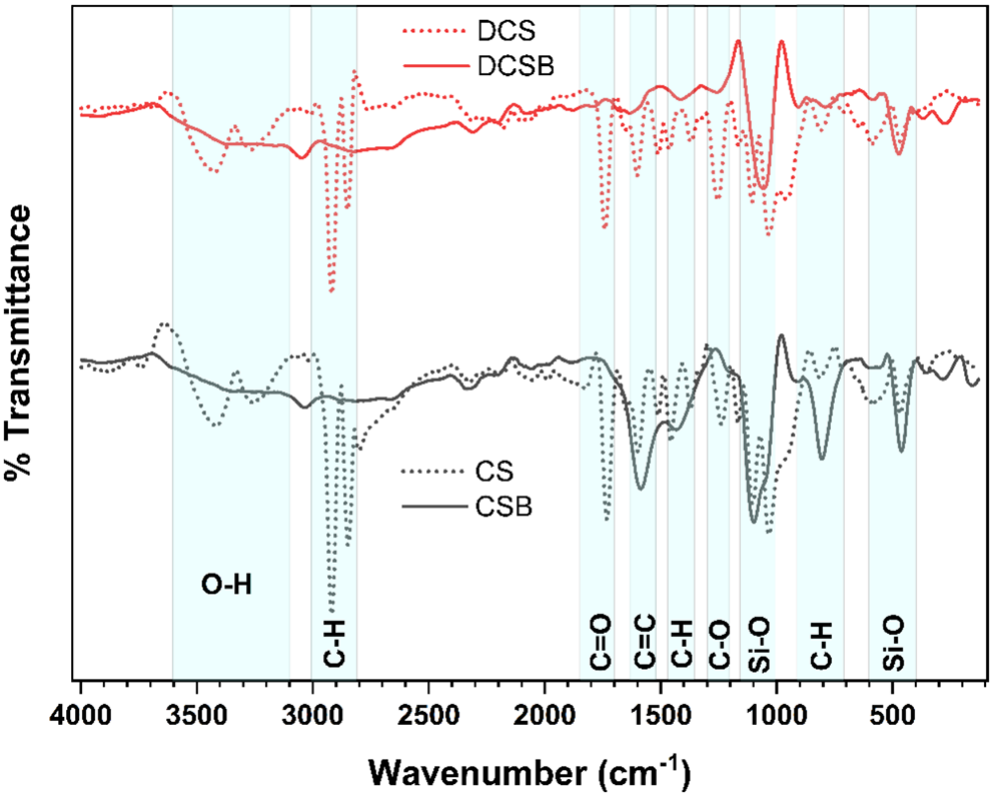
FTIR spectra of CS, DCS, CSB and DCSB.

The bands at 2917–2919 and 2844–2850 cm^–1^ indicate aliphatic C–H stretching vibrations.^47,49^ The absorption around 1732–1740 cm^–1^ is characteristic of C=O stretching of carbonyl groups in hemicellulose, such as ketones, esters, and aldehydes.^47,48^ The bands at 1646–1655 and 1596–1600 cm^–1^ are attributed to aromatic C=C and conjugated C=O stretching, typically linked to lignin.^50,51^

Vibrations at 1509–1511 and 1455–1462 cm^–1^ correspond to the aromatic skeletal vibrations of lignin and C–H deformations.^47^ Peaks at 1373–1375 and 1323 cm^–1^ reflect CH_2_ deformations and C–O vibrations in cellulose.^47^ Between 1163 and 1102 cm^–1^, bands associated with C–O–C and C–O stretching in polysaccharides, particularly cellulose and hemicellulose, are observed.^48,49^ Absorptions at 813, 714, 590, and 463 cm^–1^ are assigned to out-of-plane aromatic deformations and mineral contributions such as Si–O.^52,53^

In the biochar samples, a significant reduction in the intensity of oxygenated functional groups was observed, evidencing the deoxygenation promoted by pyrolysis. The disappearance or weakening of bands around ∼ 1730 cm^–1^ and within the 1240–1150 cm^–1^ region confirms the degradation of hemicellulose and cellulose.^48,54^

The persistence of signals between 1584 and 1636 cm^–1^ indicates the formation of more condensed aromatic structures, leading to enhanced biochar recalcitrance.^50,52^ Residual bands at ∼1415–1429 and 1255 cm^–1^ suggest the presence of lignin fragments and phenolic groups.^51,55^ Peaks within 910–804 cm^–1^ are indicative of substituted aromatic structures.^49^ Furthermore, the occurrence of bands at 576, 472, and even 371–273 cm^–1^ is associated with inorganic minerals such as silica and silicates, which remain after carbonization.^53,56^

### 3.7 Thermal Analysis

The TG/DTG and DTA curves are shown in Figure 6a–d, and thermal events are summarized in Table 3.

**Figure 6:**
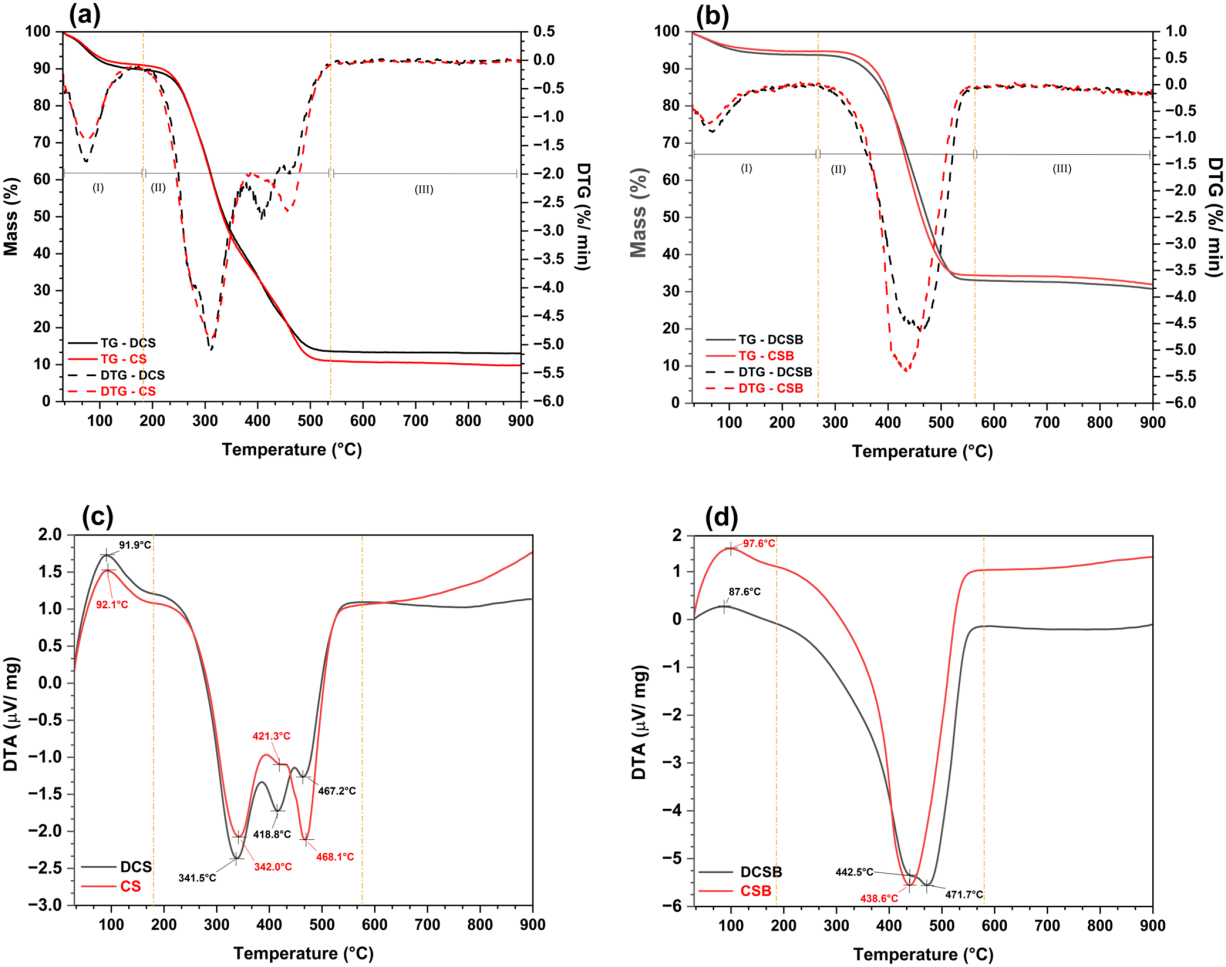
Thermograms (a) TG/DTG curves of biomasses; (b) TG/DTG curves of biochars; (c) DTA curves of biomasses and (d) DTA curves of biochars.

**Table 3:** Thermal Degradation Events and Characteristic Temperatures of CS, DCS, CSB, and DCSB Obtained from TG/DTG/DTA Analysis

	**region I**			**region II**			**region III**		
**sample**	**Δ (%)** ^a^	***T*_max_ (°C)** ^b^	***V***_**max**_ **(% min** ^–**1**^ **)** ^c^	**Δ (%)** ^a^	***T*_max_ (°C)** ^b^	***V***_**max**_ **(% min** ^–**1**^ **)** ^c^	**Δ (%)** ^a^	***T*_max_ (°C)** ^b^	***V***_**max**_ **(% min** ^–**1**^ **)** ^c^
**CS**	8.69	72.5	1.38	80.52	310.9	4.88	0.99		
**DCS**	10.05	72.5	1.77	76.43	311.0	5.09	0.49		
**CSB**	5.30	68.5	0.72	60.43	435.6	5.42	2.15		
**DCSB**	6.07	68.5	0.88	60.43	459.3	4.62	2.31		

a Δ*i*ndicates the percentage of mass loss.

b Maximum degradation temperature (°C).

c Maximum reaction rates (% min^–1^)

In the first region (30–198 °C), the biomasses CS and DCS (Figure 6a) exhibited an initial weight loss of ∼8.69 and 10.05%, with maximum degradation peaks occurring at 72.5 °C. This stage is attributed to the evaporation of water physically bound to the lignocellulosic matrix, a phenomenon related to the innate hydrophilic character of plant fibers and the release of light volatile compounds.^57,58^ Similar studies have shown that the high binding energy of water within this structure contributes to this event.^57^

The second region (198–572 °C) represented the major decomposition event for the biomasses, with intense mass losses of 80.52% for CS and 76.43% for DCS, and maximum reaction rates of 5.09% min^–1^. The DTG curves revealed overlapping peaks corresponding first to the degradation of hemicellulose (220–315 °C) and cellulose (315–400 °C), followed by the slow decomposition of lignin, which extends to higher temperatures.^58–60^ The CS and DCS presented maximum degradation rates around 311 °C. This behavior is consistent with previous reports, which indicate that hemicellulose is the most thermally labeled fraction, followed by cellulose, while lignin decomposes more gradually and contributes to char formation.^59,60^

The third region (572–900 °C) did not show pronounced peaks but only a gradual weight loss of less than 1%, attributed to residual lignin decomposition and the stabilization of the carbonaceous structure.^58,60^

For the CSB and DCSB (Figure 6b), the thermal profile was markedly different, showing higher stability. The first region showed only shallow peaks (mass losses of 5.30 and 6.07%, respectively), which are related to residual moisture and volatiles remaining after the carbonization process.^61,62^ In the second region (198–619 °C), elongated peaks were observed, with mass losses of approximately 60.43% for both. These peaks were initially associated with the decomposition of remaining cellulose, followed by the degradation of lignin.^62,63^ Notably, their maximum degradation rates shifted to significantly higher temperatures: 435.6 °C for CSB and 459.3 °C for DCSB. The third region (572–900 °C) presented a slightly higher but still gradual weight loss (2.15% for CSB and 2.31% for DCSB) and no distinct events, indicating the stabilization of the carbon matrix.^63^ Compared to the biomasses, CSB and DCSB exhibited lower overall weight loss and higher thermal stability, as expected after pyrolysis.^60,62^

The results from proximate analysis agree with the thermal behavior observed in TG/DTG and DTA. The biomasses exhibited a high volatile matter content (∼70%) and low ash content (∼12–14%), which explains the intense mass loss during the second region of decomposition, where hemicellulose and cellulose are degraded, as also reflected by the sharper DTG peaks.^58–60^

In contrast, the biochar samples presented significantly lower volatile fractions (19–35%) and higher ash contents (40–55%). These characteristics justify their reduced mass loss and the broader, less intense peaks observed in the thermal profiles, particularly in the second region (198–619 °C). The higher ash fraction in the biochars also contributes to their increased thermal stability, as inorganic residues can act as stabilizing agents during pyrolysis.^60,62^ Fixed carbon contents were comparatively higher in the biochars (∼21–22%) than in the biomasses (∼7%), consistent with the stabilization of the carbon matrix indicated in the third thermal region.

The DTA curves provided complementary information on the thermal events observed. For the biomasses (Figure 6c), the first region (30–198 °C) presented positive peaks associated with endothermic reactions, attributed to the evaporation of water and decomposition of light volatiles.^60^ In the second region (198–572 °C), three sets of negative peaks were observed. The first group, elongated, was related to hemicellulose and cellulose decomposition, while the subsequent sets corresponded to lignin degradation.^58,59^ The third region (572–900 °C) did not display evident peaks, indicating stabilization of the carbonaceous residue.^58^

For the biochars (Figure 6d), the first region (30–198 °C) exhibited only shallow peaks, corresponding to residual moisture and volatile compounds.^61,62^ In the second region (198–619 °C), the curves presented elongated negative peaks, attributed to the decomposition of residual cellulose and the degradation of lignin.^63^ The third region (572–900 °C) did not show further thermal events, indicating the stabilization of the carbon framework.^62,63^

## 4 Batch Adsorption Experiments

### 4.1 Effect of Biochar Dose

The effect of biochar dose on methylene blue removal is presented in Figure 7a (CSB) and Figure 7b (DCSB). For both biochars, removal efficiency exhibited a marked increase with increasing adsorbent mass, while the adsorption capacity demonstrated a decrease. This inverse trend is typical of batch systems with fixed initial solute concentration, in which the higher number of available active sites leads to unsaturated surfaces and, consequently, lower uptake per unit mass.^13^

**Figure 7:**
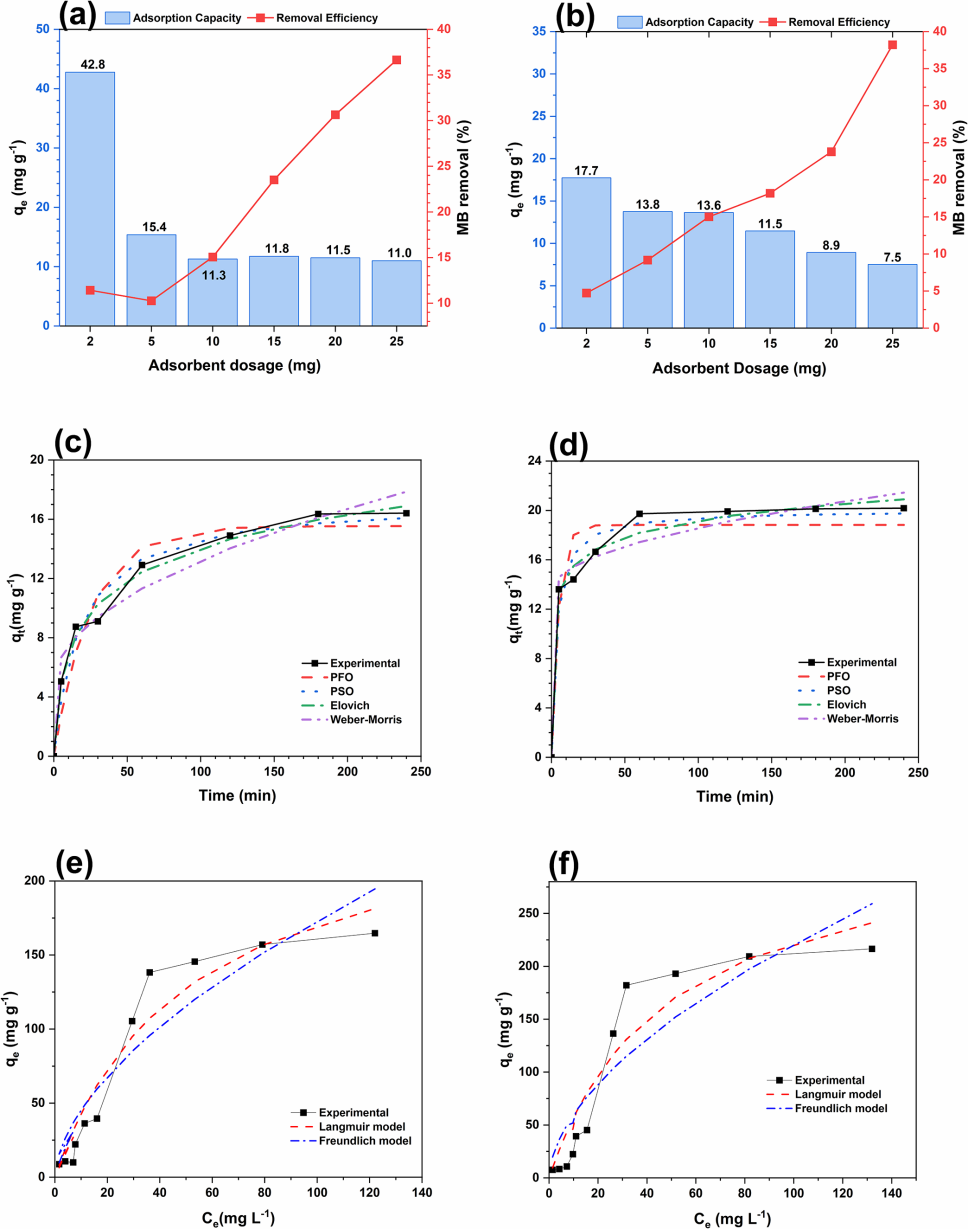
Adsorption performance of carnauba straw biochars: (a) and (b) effect of CSB and DCSB dose, respectively; (c) and (d) CSB and DCSB adsorption kinetics, respectively; (e) and (f) CSB and DCSB adsorption isotherms, respectively.

For DCSB, q_e_ decreased from 42.77 mg g^–1^ at 2 mg to 10.99 mg g^–1^ at 25 mg, accompanied by an increase in removal efficiency from 11.41 to 36.65%. CSB showed the same tendency, with q_e_ dropping from 17.73 to 7.51 mg g^–1^ across the same dosage range, while removal increased from 4.73 to 38.23%. The higher q_e_ values for DCSB at low dosages indicate a more efficient use of its active sites under conditions of high dye-to-adsorbent ratio, and the lower q_e_ of CSB suggests limited site accessibility. This behavior is consistent with reports showing that specific uptake increases at low dosages due to greater availability of dye molecules per site and decreases at higher dosages because of site overlap and insufficient solute to saturate the surface.^6^

### 4.2 Effect of Contact Time and Kinetics Models

The adsorption of methylene blue increased rapidly during the first 60 min for both CSB and DCSB (Figure 7c,d, respectively), reflecting the dominance of fast external mass transfer and the abundance of available active sites. After this stage, the uptake rate gradually slowed as surface sites became occupied, and equilibrium was reached at approximately 120 min. As shown in Table 4, DCSB achieved a higher experimental equilibrium capacity (20.18 mg g^–1^) than CSB (16.41 mg g^–1^), indicating more accessible or higher-affinity adsorption sites.

**Table 4:** Parameters of Kinetics Models Applied to the Experimental Data Obtained from Biochars for MB Adsorption

**model type**	**parameter**	**DCSB**	**CSB**
**experimental**	*q*_e_ (mg g^–1^)	20.18	16.41
**pseudo-first-order (PFO)**	*q*_e_ (mg g^–1^)	18.82	15.54
	*k*_1_ (h^–1^)	0.209	0.040
	*R* ^2^	0.496	0.876
**pseudo-second-order (PSO)**	*q*_e_ (mg g^–1^)	20.02	17.28
	*k*_2_ (g mg^–1^ h^–1^)	0.0153	0.0033
	h (mg g^–1^ h^–1^)	6.12	0.97
	*R* ^2^	0.809	0.948
**Elovich**	α (mg/g h)	361.25	2.45
	β (g mg^–1^)	0.512	0.308
	*R* ^2^	0.913	0.980
**intraparticle diffusion (Weber–Morris)**	*k*_p_ (mg/g h^1/2^)	0.517	0.843
	C (mg g^–1^)	13.43	4.79
	*R* ^2^	0.804	0.925

Analysis of the kinetic models reveals that the PFO model did not adequately describe the adsorption kinetics, given the poorer R^2^ values and the mismatch between experimental and theoretical q_e_ values. Similar deviations from PFO model behavior have been reported for various biochars when the adsorption mechanism becomes more complex near equilibrium.^9^

In contrast, the PSO model yielded the best fit for both biochars, indicating that adsorption is primarily governed by chemisorption, involving electron sharing or exchange between methylene blue molecules and active sites on the biochar surface.^4,6,29,64^

The higher initial sorption rate (h) for DCSB (6.12 mg g^–1^ h^–1^) compared with CSB (0.97 mg g^–1^ h^–1^) also reflects its stronger affinity toward MB, consistent with the higher α parameter obtained from the Elovich model (Table 4). The Elovich fits (R^2^ = 0.913 for DCSB and 0.980 for CSB) further support that adsorption occurs on heterogeneous surfaces through a chemisorption-driven mechanism.^4^

The intraparticle diffusion plot revealed multilinear behavior, suggesting that adsorption proceeds through multiple stages. The nonzero intercepts (C = 13.43 mg g^–1^ for DCSB and 4.79 mg g^–1^ for CSB) show that intraparticle diffusion is not the sole rate-limiting step, with boundary-layer diffusion also contributing significantly.^9^ Such multistep behavior—initial film diffusion, followed by pore diffusion and later chemisorptive stabilization—is consistent with patterns reported in previous studies on biochar–dye systems.^4,9^

### 4.3 Effect of Initial MB Dye Concentration and Adsorption Isotherms

The effect of initial concentration and the adsorption isotherms are presented in Figure 7e (CSB) and Figure 7f (DCSB). Increasing the initial MB concentration resulted in higher adsorption capacities for both biochars, as the stronger concentration gradient enhances the mass-transfer driving force and improves dye diffusion toward available sites.^4,6,13^ At low concentrations, removal efficiency exhibited minimal change due to the reduced number of dye molecules relative to the accessible active sites. Conversely, higher concentrations increased the dye-to-site ratio and promoted higher q_e_ until site saturation was reached.^6,9^

As demonstrated in Table 5, the Langmuir model exhibited a superior fit for both materials. The model under discussion is predicated on the assumption of monolayer adsorption on a homogeneous surface.^4,9,29^ However, biochars derived from agricultural residues are inherently heterogeneous due to the coexistence of aromatic domains, mineral phases, and surface functional groups.^7,33,50^ It has been previously established that when the Langmuir model provides the optimal fit for the adsorption process, this indicates monolayer predominance over multilayer adsorption. The finding suggests that the adsorption process is driven by the active sites, which exhibited a comparable affinity for the adsorbate.^4,9^

**Table 5:** Parameters of Adsorption Isothermal Models Applied to the Experimental Data Obtained from Biochars for MB Adsorption

**model type**	**parameter**	**DCSB**	**CSB**
**Langmuir**	***q***_**m**_ **(mg g** ^–1^ **)**	**328.13**	**255.70**
	*K*_L_ (L mg^–1^)	0.02102	0.02000
	*R* ^2^	0.902	0.934
	*R*_L_	0.24–0.95	0.25–0.95
**Freundlich**	*K*_F_	16.17	11.81
	*n*	1.76	1.71
	*R* ^2^	0.823	0.869

The maximum adsorption capacities were 328.13 mg g^–1^ for DCSB and 255.70 mg g^–1^ for CSB, confirming the higher affinity of DCSB. CS values between 0 and 1 for both biochars further indicate favorable adsorption across the tested concentration range.^6,9,29^ The Freundlich model showed lower R^2^ values, though *n* > 1 for both materials still denotes favorable adsorption on moderately heterogeneous surfaces.^6,29^

To provide a contextual framework for understanding the performance of CSB and DCSB, Table 6 presents the adsorption capacities of other biochars for MB dye removal. However, differences in preparation methodologies and experimental conditions preclude one-to-one comparisons.

**Table 6:** Comparative Analysis of MB Adsorption Capacity of Other Biochars Reported in Literature

**adsorbent**	***T***_**pyrolysis**_ **(°C)**	**pH**	***T***_**adsorption**_ **(°C)**	***q***_**m**_ **(mg g** ^–1^ **)**	**refs**
**DCSB**	550	6	25	328.13	present study
**CSB**	550	6	25	255.70	present study
***Lantana camara L.***	600	8	30	64.99	^65^
***Banana Pseudo Stem***	300	6	30	113.64	^26^
***Rice Husk***	500	7	25	17.97	^27^
***Date Palm Leaves***	700	6	25	206.61	^66^
***Natural Lignite Coal***	NA	6.35	25	40.82	^67^

### 4.4 Proposed Mechanism of Adsorption

The superior adsorption performance of DCSB toward methylene blue (MB) results from the combined contribution of multiple mechanisms typically involved in dye–biochar interactions­(Figure 8).^4,13^ Despite CSB exhibiting slightly higher O/C, its surface chemistry is mainly composed of aliphatic (C–H), unsaturated (C=C), and Si–O vibrations, offering limited polarity and a weaker interaction potential. Conversely, the lower H/C ratio of DCSB (0.43) indicates higher aromatic condensation, which promotes π–π stacking with the aromatic rings of MB, one of the dominant mechanisms governing the adsorption of cationic dyes.^4^

**Figure 8:**
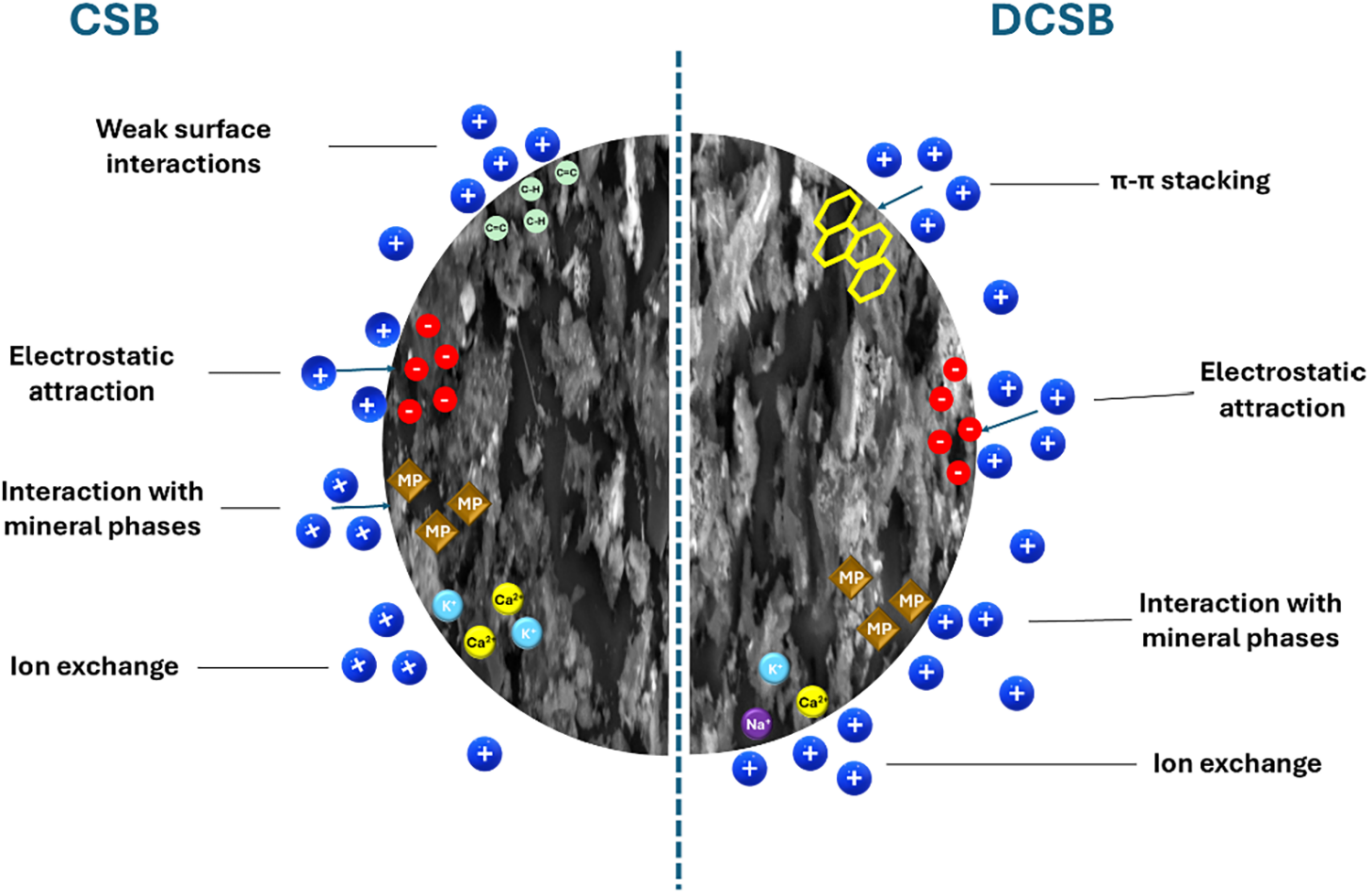
Proposed adsorption mechanism of methylene blue (MB) on CSB and DCSB biochars. Blue spheres represent cationic MB molecules.

Morphological features also play a decisive role. SEM images show that both samples display agglomerated, partially collapsed structures; however, DCSB presents a more compact and restructured carbon matrix, with fewer open voids. This densification generates more coherent adsorption domains, enhancing physicochemical interactions such as π–π stacking and surface complexation.^2,4,13^

Mineralogical characteristics further strengthen DCSB’s performance. Slightly higher levels of Si, Ca, and S detected by EDS increase surface heterogeneity, enabling electrostatic attraction, ion exchange, and mineral-assisted complexation. XRD confirms quartz, calcite, and alkali salts in both samples, but the more stabilized carbon framework of DCSB may supports more effective participation of these inorganic phases in MB binding.^2,13^

Although CSB also removes MB from the solution, its weaker performance arises from the predominance of low-polarity functional domains (C–H and C=C) and less structurally organized adsorption sites.^2,4,13^ The more open and loosely arranged carbon matrix limits the density of effective interaction domains, reducing the strength of π–π interactions and restricting the extent of electrostatic attraction and ion-exchange processes contributed by the mineral fraction. As a result, CSB relies mostly on weak van der Waals interactions and limited mineral-assisted processes, which collectively yield lower adsorption efficiency compared to DCSB.

## 5 Conclusions

The present study demonstrates the potential of CSB and DCSB biochars for the removal of methylene blue from aqueous solutions. The two materials exhibited mineral-rich surfaces and typical biochar functional groups, while DCSB showed greater aromatic condensation and a more compact structure. Adsorption experiments revealed that DCSB achieved superior performance, with higher capacity and faster kinetics. The pseudo-second-order model provided the most adequate description of the adsorption rate, and the Langmuir isotherm provided the best fit for equilibrium data, indicating monolayer chemisorption on energetically favorable sites. The combined evidence suggests that π–π interactions, electrostatic attraction, hydrogen bonding, ion exchange, and pore filling contribute to MB adsorption. In summary, DCSB is an efficient and cost-effective adsorbent that is comparable to or exceeds several reported biochars, thereby highlighting its suitability for dye removal in wastewater treatment.

## Data Availability

The data underlying this study are available in the published article and are also openly available in the Mendeley Data repository at 10.17632/rtr3797sns.1.
